# Long-Term Outcomes of a Web-Based Diabetes Prevention Program: 2-Year Results of a Single-Arm Longitudinal Study

**DOI:** 10.2196/jmir.4052

**Published:** 2015-04-10

**Authors:** S Cameron Sepah, Luohua Jiang, Anne L Peters

**Affiliations:** ^1^University of California, San FranciscoDepartment of PsychiatrySan Francisco, CAUnited States; ^2^Omada HealthSan Francisco, CAUnited States; ^3^University of California, IrvineDepartment of EpidemiologyIrvine, CAUnited States; ^4^University of Southern CaliforniaKeck School of MedicineLos Angeles, CAUnited States

**Keywords:** prediabetes, type 2 diabetes, obesity, diabetes prevention program, internet, online, mobile apps, mhealth, digital health, intervention

## Abstract

**Background:**

Digital therapeutics are evidence-based behavioral treatments delivered online that can increase accessibility and effectiveness of health care. However, few studies have examined long-term clinical outcomes of digital therapeutics.

**Objective:**

The objective of this study was to conduct a 2-year follow-up on participants in the Internet-based *Prevent* diabetes prevention program pilot study, specifically examining the effects on body weight and A1c, which are risk factors for diabetes development.

**Methods:**

A quasi-experimental research design was used, including a single-arm pre- and post-intervention assessment of outcomes. Participants underwent a 16-week weight loss intervention and an ongoing weight maintenance intervention. As part of the program, participants received a wireless scale, which was used to collect body weight data on an ongoing basis. Participants also received A1c test kits at baseline, 0.5 year, 1 year, and 2-year time points.

**Results:**

Participants previously diagnosed with prediabetes (n=220) were originally enrolled in the pilot study. A subset of participants (n=187) met Centers for Disease Control and Prevention (CDC) criteria for starting the program (starters), and a further subset (n=155) met CDC criteria for completing the program (completers) and were both included in analyses. Program starters lost an average of 4.7% (SD 0.4) of baseline body weight after 1 year and 4.2% (SD 0.8) after 2 years, and reduced A1c by mean 0.38% (SD 0.07) after 1 year and 0.43% (SD 0.08) after 2 years. Program completers lost mean 4.9% (SD 0.5) of baseline body weight after 1 year and 4.3% (SD 0.8) after 2 years, and reduced A1c by 0.40% (SD 0.07) after 1 year and 0.46% (SD 0.08) after 2 years. For both groups, neither 2-year weight loss nor A1c results were significantly different from 1-year results.

**Conclusions:**

Users of the *Prevent* program experienced significant reductions in body weight and A1c that are maintained after 2 years. Contrary to the expected progression from prediabetes to diabetes over time, average A1c levels continued to show an average regression from within the prediabetic range (5.7%-6.4%) initially to the normal range (<5.7%) after 2 years. Further investigation is warranted to test digital therapeutics as a scalable solution to address national diabetes and cardiovascular disease prevention efforts.

## Introduction

Prediabetes, the clinical precursor to type 2 diabetes, continues to grow to epidemic levels. Recent estimates by the Centers for Disease Control and Prevention (CDC) indicate that prevalence increased 8% in the last decade—from 29% in 1999-2002 to 37% in 2009-2012—amounting to 86 million Americans over age 20 with prediabetes [1,2]. Furthermore, the US Preventive Services Task Force (USPSTF) now recommends that overweight/obese adults with additional cardiovascular risk factors such as prediabetes receive intensive behavioral counseling interventions such as the Diabetes Prevention Program (DPP) [3]. As a result, there is an urgent need for effective chronic disease prevention interventions that can be disseminated to millions of people. To address such needs, a new field of “digital therapeutics” has emerged—evidence-based treatments from the field of behavioral medicine that are delivered online [4].

One of the most established digital therapeutics is *Prevent*, an Internet-based version of the DPP clinical trial. Though the DPP was primarily an individual treatment, most DPP translations including *Prevent* have successfully used group-based approaches to minimize cost, and thus group-based interventions have been recommended in evidence-based guidelines for diabetes prevention [5].

Previous published research showed that *Prevent* program starters were able to achieve clinically significant reductions in body weight and A1c after 1 year [6]. Of note, this was the first study to show that a scalable Internet-based intervention met all efficacy benchmarks for DPP programs set by CDC’s Diabetes Prevention and Recognition Program (DPRP) standards [7]. This addressed a key concern raised by Yudkin & Montori that “rolling out intensive lifestyle interventions like these to populations with pre-diabetes…would be challenging” [8].

Furthermore, a critique of DPP studies by Kahn and Davidson noted that weight loss results of DPP translations have often been low (a meta-analysis of 22 studies showing 2.4% weight loss at 1 year) [9] and often show significant weight regain after the intervention ends. However, they concede that real-world DPPs “may have promise if the weight loss achieved at year 2 can be sustained and replicated” [10].

Thus, the current study seeks to make an original contribution to the scientific literature by addressing such points through investigating the long-term outcomes and sustainability of an Internet-based DPP.

## Methods

### Research Design

A quasi-experimental research design was used, including a single-arm pre- and post-intervention assessment of body weight, A1c, and program engagement outcomes [6]. Participants were not compensated for participation but were enrolled in the program at no cost. Institutional Review Board (IRB) exemption was granted by Western IRB for secondary analyses of previously collected and de-identified data.

### Participants

Patients were recruited via craigslist advertisements seeking participants for an Internet-based diabetes prevention program. Patients were screened for a self-reported clinical diagnosis of prediabetes within the past year and meeting CDC DPRP eligibility criteria: 18 years of age or older, have a body mass index (BMI) of ≥24 kg/m^2^ (≥22 kg/m^2^ if Asian), and able to engage in light physical activity [7].

Eligible participants completed an online account set-up process, in which they provided consent and completed health and demographic questionnaires, and then enrolled in the *Prevent* program, which they could access via any Internet-enabled desktop or mobile device [11].

### Program

A full description of the program components has been previously published [6]. Briefly, *Prevent* is an Internet-based translation of the DPP lifestyle intervention, which includes small group support, personalized health coaching, a weekly DPP-based curriculum, and digital tracking tools. Participants were demographically matched into groups of 10-15 participants and placed into a private online social network resembling Facebook where they could discuss goal progress and provide social support to one another. At any convenient time or place using Internet-enabled devices, they could asynchronously complete weekly DPP-based health education lessons, privately message and call a health coach for individual counseling, track weight loss, and physical activity using a wireless weight scale and pedometer, and monitor their engagement and weight loss progress.

Prevent starts with a 16-week “Core” curriculum focusing on weight loss and continues with a 36-week post-core “Sustain” curriculum focusing on weight maintenance, with this “active” intervention totaling 12 months. During this time, participants also engage with health coaching, small group discussion, and tracking of body weight/food/physical activity. After 12 months, participants continue with an ongoing intervention that is more proactive, in which they continue to have access to past curriculum, a larger *Prevent* “alumni” group discussion forum, and tracking capabilities.

### Measures

Baseline demographic and health information were collected prior to program start using an Internet-based questionnaire self-completed by the subject. Body weight, the study’s primary outcome, was measured serially in pounds using a validated, wireless-enabled scale that was mailed to participants [12]. Weights were transmitted over a 2G cellular network to a central study server. Participants were encouraged to weigh themselves daily and reminded via email/telephone calls to obtain weights at the baseline, 6, 12, and 24-month assessment timepoints. Weight measurements were highly stable and the scale’s coefficient of variation was ±0.2 lb.

A1c was measured in percent (NGSP/DCCT units) using self-administered AccuBase A1c test kits by DTI laboratories, an FDA-cleared whole blood test that uses a capillary tube blood collection method. This allows for reliable home-based data collection and valid lab testing using high-performance liquid chromatography (HPLC-IE/HPLC-BA), including abnormal hemoglobin screening [13]. A1c test kits were mailed to participants’ homes around the baseline, 6, 12, and 24-month assessment timepoints.

### Analyses

Results were analyzed for two subgroups based on CDC DPRP standards: “program starters” were those who completed at least 4 core lessons, and “program completers” were those who completed as least 9 core lessons.

Results were analyzed using SPSS Statistics 21.0 and SAS 9.3. Baseline characteristics were compared between subgroups using chi-square tests or Fisher’s exact test for categorical variables and two-sample *t* tests for continuous variables. To account for repeated measures and missing data, linear mixed-effects models were used to obtain adjusted mean changes in weight and A1c over the 2-year follow-up period. Based on exploratory data analysis and graphs of the time trends, piecewise linear models were fit for weight change with days from baseline and a change point after the last core lesson day included in the model. For A1c, an additional change point at 12 months was added to the mixed model due to significant change in slope at that timepoint and better model fit statistics.

Models used an autoregressive-moving-average covariance structure to statistically account for the correlation of frequently measured weight data. Repeated A1c measures were also correlated but not measured on a daily basis. Therefore a spatial power covariance structure (with time as the distance measure) was used to account for the correlation among repeated measures of A1c from the same participant.

## Results

### Demographics and Participation

**Figure 1:**
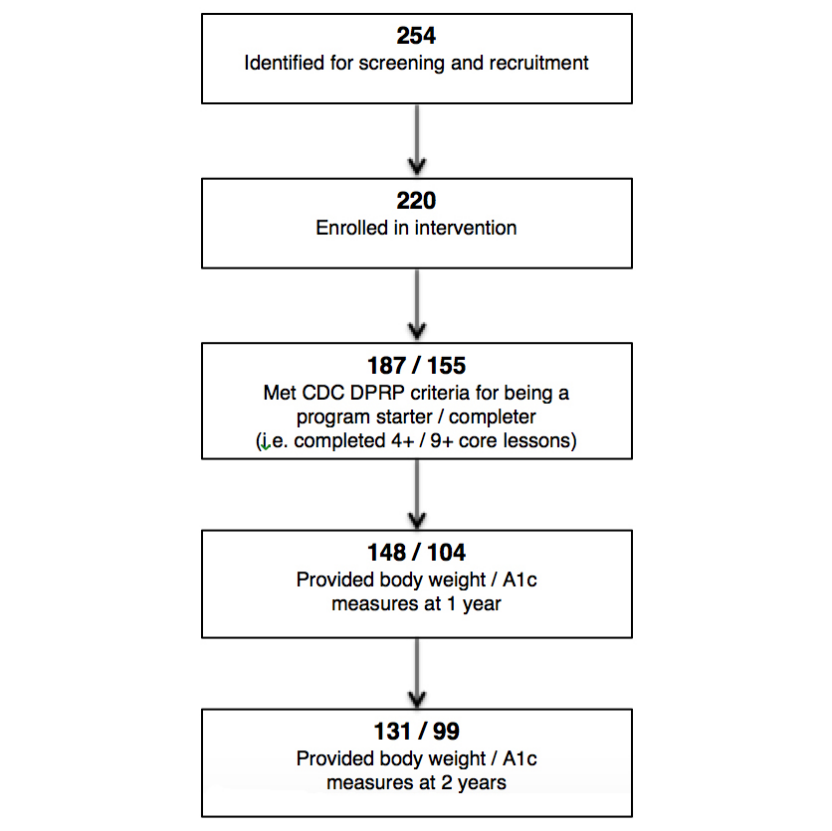
Participant recruitment and retention flow chart.

As shown in Figure 1, 254 participants from across the United States responded to online advertisements and met CDC DPRP eligibility criteria. Of these, 220 participants completed the initial assessment and online set-up process and enrolled in the intervention on April 29, 2012. Demographic characteristics of study participants are reported in a previous study [5]. Briefly, as outlined in Table 1, the participants were socioeconomically diverse. The baseline BMI of these 220 participants was 36.6 kg/m^2^. A subset of these participants (n=187) completed at least 4 core lessons and were thus designated program starters, and a further subset of these participants (n=155) completed at least 9 core lessons and were thus designated program completers.

**Table 1:** Baseline demographic characteristics of participants.

Characteristics		Total (N=220)	Non-starters (0-3 lessons) (n=33)	Starters (4+ lessons) (n=187)	Non-starters vs Starters, *P* value	Non-completers (4-8 lessons) (n=32)	Completers (9+ lessons) (n=155)	Non-completers vs Completers, *P v*alue
Age, mean (SD)		43.6 (12.4)	42.0 (12.6)	43.9 (12.4)	.43^a^	39.0 (9.4)	44.9 (12.8)	.004^a^
Weight, mean (SD)		223.1 (47.9)	226.1 (53.5)	222.5 (47.0)	.69^a^	229.8 (45.9)	221.0 (47.2)	.34^a^
BMI, mean (SD)		36.6 (7.5)	35.9 (6.6)	36.7 (7.6)	.56^a^	38.3 (7.5)	36.4 (7.6)	.21^a^
Gender (male), n (%)		38 (17.3)	10 (30.3)	28 (15.0)	.03^b^	3 (9.4)	25 (16.1)	.42^c^
**Ethnicity, n (%)**					.28^b^			.89^c^
	White	108 (50.2)	15 (45.5)	93 (51.1)		15 (48.4)	78 (51.7)	
	Black	63 (29.3)	10 (30.3	53 (29.1)		11 (35.5)	42 (27.8)	
	Hispanic	23 (10.7)	2 (6.1)	21 (11.5)		3 (9.7)	18 (11.9)	
	Other	21 (9.8)	6 (18.2)	15 (8.2)		2 (6.5)	13 (8.6)	
**Marital status, n (%)**					.13^c^			.68^c^
	Married/live with a partner	87 (57.6)	9 (50.0)	78 (58.6)		15 (68.2)	63 (56.8)	
	Divorced/separated/widowed	25 (16.6)	1 (5.6)	24 (18.1)		3 (13.6)	21 (18.9)	
	Never married	39 (25.8)	8 (44.4)	31 (23.3)		4 (18.2)	27 (24.3)	
**Education, n (%)**					.01^b^			.13^b^
	< College graduate	72 (48.3)	14 (77.8)	58 (44.3)		13(59.1)	45 (41.3)	
	≥ College graduate	77 (51.7)	4 (22.2)	73 (55.7)		9 (40.9)	64 (58.7)	
**Income (USD), n (%)**					.92^b^			.52^b^
	<$50,000	69 (48.3)	8 (47.1)	61 (48.4)		11 (55.0)	50 (47.2)	
	$50,000 or higher	74 (51.8)	9 (52.9)	65 (51.6)		9 (45.0)	56 (52.8)	

^a^ *P* value of 2-sample *t* test.

^b^ *P* value of chi-square test unless otherwise noted.

^c^ *P* value of Fisher’s exact test.

### Engagement

As reported in a previous publication, during the first year of the program, program starters completed an average of 13.8 and 3.2 lessons during the core and post-core phases (CDC DPRP benchmark is 9 and 3 lessons), documented body weight at 90% and 67% of weeks and months in which core and post-core lessons were completed (benchmark is 80% and 60%), and documented physical activity at 85% of weeks in which core lessons were completed (benchmark is 80%) [6].

Because lessons are limited to the first year, no engagement benchmarks exist beyond 1 year. However, participants continued to weigh in an average of 6.2 (SD 0.3) of 12 months and logged in an average of 3.5 (SD 0.3) of 12 months between years 1 and 2. In order to evenly compare engagement rates between years 1 and 2, the last 8 months of the year 1 (ie, the Sustain post-core period) was compared to the first 8 months of year 2. *Prevent* program starters weighed in an average of 4.6 (SD 0.2) of the last 8 months of year 1 versus 4.5 (SD 0.2) of the first 8 months of year 2, a non-significant difference (*P*=.708). *Prevent* program starters logged in on an average of 3.5 of 8 (SD 0.3) last months of the year 1 versus 2.7 of 8 (SD 0.3) first months of year 2, which was a significant difference (*P*=.008).

### Changes in Body Weight and A1c

Among program starters, 100% (187/187) had an initial baseline weight measurement, 78.6% (147/187) had at least one weight measurement between 15 and 17 weeks, 79.1% (148/187) had at least one weight measurement between 11 and 13 months, and 70.1% (131/187) had at least one weight measurement between 22 and 26 months.

Changes in body weight and A1c after 16 weeks and 1 year were reported in a previous publication and reported here alongside 2-year data in Tables 2 and 3 [6]. From baseline to year 2, program starters and completers achieved mean weight loss of 4.2% (SD 0.8%) and 4.3% (SD 0.8%), respectively. Program starters’ and completers’ weight loss was also maintained from year 1 to year 2, with no significant change (*P*=.25 and .20, respectively). Intention-to-treat analyses were also conducted on all enrollees: from baseline to year 2, enrollees achieved an average weight loss of 4.5% (SD 0.7%), though the majority of these additional participants did not weigh in after 1 year, which contributed to a higher possibility of model error. Among the program starters who reported weight between 22 and 26 months (n=131), 52 (40.0%) continued to meet or exceeded the CDC DPRP 5% weight loss benchmark at 24 months.

Because A1c measurement was optional, compliance was lower. Among program starters, 75.4% (141/187) had an initial baseline A1c measurement, 36.4% (68/187) had an A1c measurement between months 6-8 (due to A1c being a lagging measurement), 55.6% (104/187) had an A1c measurement between months 12-14, and 52.9% (99/187) had an A1c measurement between months 24-28.

As shown in Tables 2 and 3, from baseline to year 2, program starters and completers reduced A1c by 0.43% (SD 0.08) and 0.46% (SD 0.08), respectively. Program starters’ and completers’ A1c reduction was also maintained from year 1 to year 2, with no significant change (*P*=.39 and .38, respectively).

**Table 2:** Body weight and A1c of participants over time.

	Starters (4+ lessons)		Completers (9+ lessons)	
	Weight (lbs), mean (SE)^a^	A1c (%), mean (SE)^a^	Weight (lbs), mean (SE)^a^	A1c (%), mean (SE)^a^
Baseline	221.6 (3.5)	5.99 (0.07)	220.2 (3.9)	6.02 (0.08)
16 weeks	210.5 (3.5)	6.02 (0.08)	208.6 (3.9)	6.04 (0.09)
Year 1	211.3 (3.4)	5.61 (0.08)	209.5 (3.8)	5.62 (0.08)
Year 2	212.3 (3.5)	5.55 (0.08)	210.7 (3.9)	5.56 (0.08)

^a^Adjusted means from linear mixed models.

**Table 3:** Changes in body weight and A1c of participants over time.

	Starters (4+ lessons)				Completers (9+ lessons)			
	Weight loss, % change (SE)^a^	*P* value	A1c, change (SE)^a^	*P* value	Weight loss, % change (SE)^a^	*P* value	A1c, change (SE)^a^	*P* value
16 weeks-Baseline	5.0 (0.3)	<.001	0.03 (0.06)	.55	5.2 (0.3)	<.001	0.03 (0.06)	.62
Year 1-Baseline	4.7 (0.4)	<.001	-0.38 (0.07)	<.001	4.9 (0.5)	<.001	-0.40 (0.07)	<.001
Year 2-Baseline	4.2 (0.8)	<.001	-0.43 (0.08)	<.001	4.3 (0.8)	<.001	-0.46 (0.08)	<.001
Year 2-Year 1	-0.5 (-0.4)	.25	-0.06 (0.07)	.39	-0.5 (-0.5)	.20	-0.06 (0.07)	.38

^a^Adjusted means from linear mixed models.

## Discussion

### Principal Findings

Results indicate that the *Prevent* Internet-based diabetes prevention program helped participants who started or completed the program achieve significant reductions in body weight and A1c after 2 years. Of note, weight loss was largely maintained between years 1 and 2; this is one of the few studies to exhibit this weight maintenance effect after the active intervention ended.

Furthermore, A1c continued to show an average reduction from within the prediabetes range (5.7%-6.4%) to the normal range (<5.7%), in contrast to an expected annual rate of progression of approximately 4% from prediabetes to type 2 diabetes [14]. Thus, these results address critiques regarding the ability of DPP translations to show long-term effects on weight loss and A1c reduction [7,9].

The ability of *Prevent* to show sustained effects may be due to unique aspects of the program. Although the active intervention was 12 months, participants have continued access to *Prevent* and can proactively use the intervention. Thus, by allowing ongoing health tracking and social support, digital therapeutics can promote long-term maintenance of lifestyle changes with little ongoing intervention cost or maintenance.

Although *Prevent* exceeded CDC DPRP benchmarks for program completion during the 16-week core phase, 30% of participants who initially enrolled still did not complete the program. We hypothesize that one of the key advantages of digital therapeutics—accessibility—may also be a disadvantage when it comes to program completion. The lower barrier to Internet-based program entry inevitably increases the number of participants with lower motivation, who may have never made the effort to show up to an in-person program.

### Strengths and Limitations

Study strengths include longitudinal collection of body weight and A1c data and statistical analysis using linear mixed models, which allow for more robust estimation over time and missing data. Furthermore, recruitment, intervention, and assessment were done exclusively remotely, in contrast to most digital health studies that require in-person orientation or follow-up assessment. This enhances the generalizability of the findings to “real-world” commercial deployments that must be done remotely.

Study limitations include a non-randomized, uncontrolled single-arm design with a self-selected sample, which precludes causal inference of the intervention to outcomes. However, this also better approximates how commercial programs enroll real-world populations. Fewer males participated in the study, but this is typical of behavioral weight loss interventions. Furthermore, 70% of program starters had weight data at year 2 and 53% had A1c data at year 2, which limits generalizability regarding outcomes on all participants. Thus, conclusions regarding weight loss and A1c reductions are limited to program starters and completers. In addition, while adherence to program behaviors (eg, lesson completion, tracking of weight and physical activity) were assessed according to CDC DPRP standards, adherence to health behaviors (eg, diet and exercise goals) were not, which limits causal inference.

### Conclusions

Results of this study suggest that the *Prevent* Internet-based diabetes prevention program was able to produce significant reductions in body weight and A1c that are sustained over the course of 2 years, even after active intervention ended after 1 year. Further investigation is warranted to test digital therapeutics as a scalable solution to address national diabetes and cardiovascular disease prevention efforts.

## Abbreviations

BMI: Body Mass Index
CDC: Centers for Disease Control and Prevention
DPP: Diabetes Prevention Program
DPRP: Diabetes Prevention Recognition Program
HPLC: High-Performance Liquid Chromatography
IRB: Institutional Review Board
USPSTF: US Preventive Services Task Force
